# Microbiological pathogen analysis in native versus periprosthetic joint infections: a retrospective study

**DOI:** 10.1186/s13018-021-02850-3

**Published:** 2022-01-06

**Authors:** Sebastian Linke, Alexander Thürmer, Kevin Bienger, Christian Kleber, Petri Bellova, Jörg Lützner, Maik Stiehler

**Affiliations:** 1 University Centre of Orthopaedic, Trauma and Plastic Surgery, University Hospital Carl Gustav Carus Dresden, TU Dresden, Fetscherstraße 74, 01307 Dresden, Germany; 2 Department of Medical Microbiology and Hygiene, Medical Faculty Carl Gustav Carus, TU Dresden, Dresden, Germany

**Keywords:** Joint infection, Pathogen spectrum, Synovial fluid, Prosthesis, Native

## Abstract

**Background:**

The presence or absence of an implant has a major impact on the type of joint infection therapy. Thus, the aim of this study was the examination of potential differences in the spectrum of pathogens in patients with periprosthetic joint infections (PJI) as compared to patients with native joint infections (NJI).

**Methods:**

In this retrospective study, we evaluated culture-positive synovial fluid samples of 192 consecutive patients obtained from January 2018 to January 2020 in a tertiary care university hospital. For metrically distributed parameters, Mann–Whitney U was used for comparison between groups. In case of nominal data, crosstabs and Chi-squared tests were implemented.

**Results:**

Overall, 132 patients suffered from periprosthetic joint infections and 60 patients had infections of native joints. The most commonly isolated bacteria were coagulase-negative Staphylococci (CNS, 28%), followed by *Staphylococcus aureus* (*S. aureus*, 26.7%), and other bacteria, such as Streptococci (26.3%). We observed a significant dependence between the types of bacteria and the presence of a joint replacement (*p* < 0.05). Accordingly, detections of CNS occurred 2.5-fold more frequently in prosthetic as compared to native joint infections (33.9% vs. 13.4% *p* < 0.05). In contrast, *S. aureus* was observed 3.2-fold more often in NJIs as compared to PJIs (52.2% vs. 16.4%, *p* < 0.05).

**Conclusion:**

The pathogen spectra of periprosthetic and native joint infections differ considerably. However, CNS and *S. aureus* are the predominant microorganisms in both, PJIs and NJIs, which may guide antimicrobial therapy until microbiologic specification of the causative pathogen.

## Background

Due to the increase in joint replacement operations [1], PJIs have become a relevant challenge in modern medicine [2]. Regarding NJIs, low incidences of four to ten cases per 100,000 individuals per year have been reported and a possible increase is still controversial [3, 4]. Nevertheless, both infection types share a high risk for complications, necessitating differentiated diagnostics [5, 6].

Patients suffering from PJI frequently show recurrences [7]. Prolonged hospitalization and delayed functional recovery are common consequences and possible reasons for the elevated five-year-mortality rate of up to 26% [4, 6]. NJIs are no less demanding, as they cause a significant risk for secondary osteoarthritis and permanent joint damage, leading to loss of function in about 40% of cases [6, 8].

An adequate initial empiric antibiotic therapy is therefore crucial. However, treating the predominant pathogens can be a challenge. Regardless of the infection type, blood cultures often remain inconclusive [9] and possible contamination in analyses of tissue may provide false-negative results. Awareness of the expected pathogens in periprosthetic, as well as in native joint infections could therefore accelerate diagnostic procedures and increase the patient’s outcome.

In implant-associated infections, biofilm formation is a characteristic pathomechanism. It weakens the effect of the physiological immune response and the effect of an antimicrobial therapy as well [10]. Certain bacteria, such as *Staphylococcus epidermidis* (*S. epidermidis*) and *S. aureus*, show a high capacity for biofilm formation [11]. Therefore, their detection rate in PJIs is comparatively high [12, 13]. In native joints, the emergence of an infection depends as much on the success or failure of the local and systemic immune response. Accordingly, highly potent virulence factors and signaling mechanisms characteristic of *S. aureus* increase the likelihood of infection [14]. The literature considers both CNS and *S. aureus* as predominant causatives in periprosthetic and native joint infections as well [12, 15]. Evidence for differing pathogen distributions, such as a dominance of *S. aureus* in NJIs and of CNS in PJIs, is weak [10, 16]. Therefore, it remains inconclusive, whether the presence of a joint replacement influences the type of causative bacteria in joint infections, which could necessitate different empiric antibiotic therapies. To this end, we retrospectively examined differences in the spectrum of pathogens based on synovial fluid analyses in patients with PJI as compared to those with NJI.

## Materials and methods

### Study design and endpoints

In this retrospective level 3 study, we screened all microbial synovial fluid analyses performed at our tertiary care university hospital from January 2018 to January 2020. Inclusion criteria were a positive culture result in the microbiological analysis and that the samples originated from a closed joint aspiration. We then investigated whether the patient in question had subsequently undergone surgery for a joint infection and whether laboratory markers of a joint infection were present at the time of aspiration, such as an increased CRP concentration in the serum and a raised leukocyte count in the synovial fluid. If both criteria were not fulfilled, the exclusion criteria were met.

The samples were classified depending on the presence or absence of a prosthesis in the infected joint. Intergroup differences in the pathogen spectrum of synovial fluid samples were the primary endpoint. In addition, the infection sites and the number of mixed infections were assessed. Age, gender, previous immunosuppressive therapy, diabetes mellitus or renal insufficiency were analyzed in the context of patient characteristics. Due to the anonymized data collection and the retrospective design, no ethics application was necessary for the study conduct according to Saxonian legislation.

### Defined categories of isolated pathogens

The spectrum of isolated pathogens was stratified into five groups, based on clinical aspects and the number of detections. The group of CNS included *S. epidermidis*, *S. capitis*, *S. hominis*, *S. lugdunensis* and *S. caprae*. Since it is the only coagulase-positive Staphylococcus and because of its high detection rate, we defined *S. aureus* a single pathogen class. Within the group of Enterobacterales, we subsumed the detected species of *E. coli*, *Enterobacter cloacae* complex, *Proteus* spp. and *Klebsiella* spp.. Obligatory anaerobic pathogens were *Anaerococcus* spp., *Bacteroides fragilis*, *Clostridium difficile* and *Fusobacterium mortiferum*. The last group consists of other pathogens, such as different species of *Enterococci* and *Streptococci*, *Pseudomonas aeruginosa*, and other, rarely detected microorganisms.

### Statistics

With respect to the patients’ age being a metrically distributed parameter, a non-normal distribution was proven using the Shapiro–Wilk test, and Mann–Whitney U was applied for intergroup comparison. For nominal data, such as the infection sites and the isolated pathogen types, crosstabs and Chi-squared tests were implemented using the contingency coefficient (*C*) to confirm significant differences. Regarding associations between the presence of renal insufficiency or diabetes mellitus and possible differences in the pathogen distribution, a binary logistic regression analysis was performed. In general, the confidence interval was set to 95%.

## Results

### Study population and patient characteristics

During the observation period of 2 years, 192 patients were diagnosed culture-positive by synovial fluid analyses (Table 1). Of these, 132 had periprosthetic joint infections and 60 suffered from native joint infection. On average, patients with PJI were significantly older as compared to patients with NJI (72 vs. 59.5 years, *p* < 0.01). However, no differences in gender distribution occurred.

**Table 1:** Study population, patient characteristics, microbial detection rates, and infection sites

	PJI	NJI	*P*	Total
*Patients (n [%])*	132 [68.8]	60 [31.2]		192 [100]
*Patient characteristics*				
Age (median ± SD)	72 ± 12.1	59.5 ± 24.7	< 0.01	66.3 ± 18.4
Sex				
Male (n [%])	68 [51.5]	40 [66.7]		108 [56.3]
Female (n [%])	64 [48.5]	20 [33.3]		84 [43.7]
Immunosuppression (n [%])	11 [8.3]	8 [13.3]		19 [9.9]
Diabetes mellitus (n [%])	41 [31.1]	10 [16.7]	0.036	51 [26.6]
Renal insufficiency (n [%])	39 [29.5]	12 [20]		51 [26.6]
*Microbial detection rates*				
Pathogen detections (n [%])	165 [71.1]	67 [28.9]		232 [100]
Mixed infections (n [%])	33 [25]	7 [11.7]		40 [20.8]
*Infection sites*				
Hip	86 [65.2]	10 [16.7]	< 0.01	96 [50]
Knee	39 [29.5]	37 [61.7]	< 0.01	76 [39.6]
Shoulder	1 [0.75]	6 [10]		7 [3.6]
Ankle	4 [3]	2 [3.3]		6 [3.1]
Elbow	1 [0.75]	5 [8.3]		6 [3.1]
Wrist	1 [0.75]	0 [0]		1 [0.5]

*SD* standard deviation

In the cohort investigated, the most common localization of joint infection was the hip (50%), followed by the knee (39.5%), and the shoulder (3.6%) joint. Regarding joint replacements, infections occurred more frequently in the hip (65.2%) compared to the knee (29.5%) joint. An opposite result was observed in native joints with 61.7% of infections being detected in the knee, and 16.7% in the hip joint. Overall, 19 patients received immunosuppressive treatment with a rate independent of the presence of an endoprosthesis. The rate of diabetes mellitus was significantly higher in the PJI group as compared to the NJI group (31.1% vs. 16.7%, *p* = 0.036), while no differences occurred regarding the existence of renal insufficiency.

### Pathogen spectrum in synovial fluid analyses

In 192 samples, we isolated 232 pathogens with 165 and 67 of them belonging to the PJI and the NJI group, respectively (Table 1). Hence, 33 (25%) mixed infections occurred in periprosthetic joints and seven (11.7%) in native joints. In total, 32 different pathogens were isolated. Staphylococcal species were by far the most frequently detected pathogens with 83 (50.3%) positive results in the PJI and 44 (65.7%) in the NJI group (Table 2). With a detection rate of 62/232 (26.7%), *S. aureus* was the most commonly isolated pathogen, followed by *S. epidermidis*, being identified in 48 cases (20.7%). There was one case of oxacillin-resistant *S. aureus* (MRSA) in the overall study population, detected in a PJI patient.

**Table 2:** Total pathogen spectrum in PJIs and NJIs (n [%])

Pathogen	PJI (N = 165)	NJI (N = 67)	Total (N = 232)
*Staphylococcus* spp.	83 [50.3]	44 [65.7]	127 [54.7]
* Oxacillin-resistant Staphylococcus spp.*	38 [45.8*]	4 [9.1*]	42 [33.1*]
*Staphylococcus aureus*	27 [16.4]	35 [52.2]	62 [26.7]
* Oxacillin-resistant Staphylococcus aureus*	1 [3.7*]	0 [0*]	1 [1.6*]
Coagulase-negative staphylococci	56 [33.9]	9 [13.4]	65 [28]
* Oxacillin-resistant CNS*	37 [66.1*]	4 [44.4*]	41 [63.1*]
* Staphylococcus epidermidis*	44 [26.7]	4 [6]	48 [20.7]
* Staphylococcus capitis*	4 [2.4]	2 [3]	6 [2.6]
* Staphylococcus hominis*	3 [1.8]	2 [3]	5 [2.2]
* Staphylococcus lugdunensis*	3 [1.8]	1 [1.5]	4 [1.7]
* Staphylococcus caprae*	2 [1.2]	0 [0]	2 [0.9]
Enterobacteriaceae	31 [18.8]	5 [7.5]	36 [15.5]
*Escherichia coli*	14 [8.5]	3 [4.5]	17 [7.3]
*Enterobacter cloacae* complex	11 [6.7]	1 [1.5]	12 [5.2]
*Klebsiella* spp.	2 [1.2]	1 [1.5]	3 [1.3]
*Proteus* spp.	4 [2.4]	0 [0]	4 [1.7]
Obligatory anaerobic pathogens	7 [4.2]	1 [1.5]	8 [3.4]
* Bacteroides fragilis*	3 [1.8]	0 [0]	3 [1.3]
* Anaerococcus* spp.	2 [1.2]	0 [0]	2 [0.9]
*Clostridium difficile*	1 [0.6]	1 [1.5]	2 [0.9]
* Fusobacterium mortiferum*	1 [0.6]	0 [0]	1 [0.4]
Other pathogens	44 [26.7]	17 [25.4]	61 [26.3]
* Enterococcus faecalis*	8 [4.8]	4 [6]	12 [5.2]
* Pseudomonas aeruginosa*	7 [4.2]	1 [1.5]	8 [3.4]
* Streptococcus dysgalactiae*	4 [2.4]	4 [6]	8 [3.4]
* Propionibacterium acnes*	6 [3.6]	1 [1.5]	7 [3]
* Streptococcus agalactiae*	4 [2.4]	0 [0]	4 [1.7]
* Enterococcus faecium*	4 [2.4]	0 [0]	4 [1.7]
* Streptococcus pneumoniae*	0 [0]	3 [4.5]	3 [1.3]
* Streptococcus gallolyticus*	3 [1.8]	0 [0]	3 [1.3]
* Streptococcus oralis*	1 [0.6]	1 [1.5]	2 [0.9]
* Streptococcus pyogenes*	1 [0.6]	1 [1.5]	2 [0.9]
* Acinetobacter baumannii*	0 [0]	1 [1.5]	1 [0.4]
* Candida albicans*	1 [0.6]	0 [0]	1 [0.4]
* Listeria monocytogenes*	1 [0.6]	0 [0]	1 [0.4]
* Morganella morganii*	0 [0]	1 [1.5]	1 [0.4]
* Streptococcus anginosus*	1 [0.6]	0 [0]	1 [0.4]
* Streptococcus constellatus*	1 [0.6]	0 [0]	1 [0.4]
* Streptococcus salivarius*	1 [0.6]	0 [0]	1 [0.4]
* Streptococcus sanguinis*	1 [0.6]	0 [0]	1 [0.4]

*PJI* periprosthetic joint infection, *NJI* native joint infection

Percentages relate to total detections of the respective group (PJI, NJI and Total)

^*^Percentages relate to the value of the line above

**Fig. 1:**
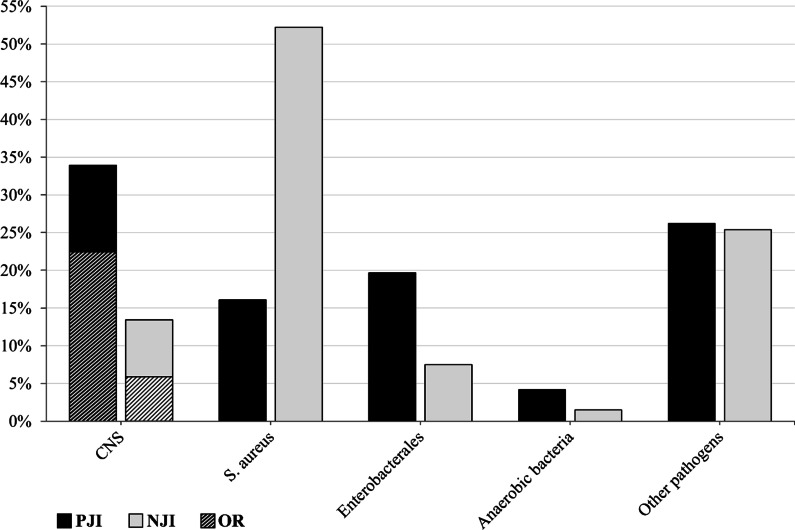
Detection rates of pathogen classes in PJI and NJI. PJI: Periprosthetic joint infection. NJI: Native joint infection. OR: Oxacillin resistance. X-axis: pathogen classes. Y-axis: detection rates

Infections caused by *S. aureus* were 3.2-fold more frequent in NJIs (52.5%) as compared to PJIs (16.4%, CI 95%). Contrarily, infections with *S. epidermidis* occurred 4.5-fold more often in PJIs (26.7%) as compared to NJIs (6%, CI 95%). In general, there were significant differences in the distribution of pathogen types, depending on the presence or absence of a prosthesis in the infected joint (*C* = 0.47, *P* < 0.05). With reference to the pathogen classes, CNS (*n* = 65, 28%) were most common (Fig. 1), followed by *S. aureus* (see above), other pathogens (*n* = 61, 26.3%) and Enterobacterales (*n* = 36, 15.5%). Infections with CNS occurred more frequently in PJIs (*n* = 56, 33.9%) as compared to NJIs (*n* = 9, 13.4%). Of these CNS detections, 37 (66.1%) and four (44.4%) were oxacillin-resistant in the PJI and NJI group, respectively, without a significant intergroup difference regarding their detection rate.

Furthermore, we detected a 2.5-fold increased rate of infections with Enterobacterales in periprosthetic joints (*n* = 31, 18.8%) as compared to native joints (*n* = 5, 7.5%, CI 95%). The differences regarding anaerobic and other pathogens were minor. Overall, the distribution of pathogen classes differs significantly between PJIs and NJIs (*C* = 0.363, *p* < 0.05).

There was no significant between the presence of renal insufficiency or diabetes mellitus and the pathogen spectrum in the entire study population as well as in the subgroups.

## Discussion

In this study, we detected significant differences in the pathogen spectrum of PJIs as compared to NJIs. *Staphylococcus aureus* was isolated 3.2 times more often in infections of native joints. Contrarily, infections with CNS were 2.5 times more common in PJIs. Besides staphylococcal species, we also found a 2.5-fold increased detection rate of Enterobacterales in prosthetic as compared to native joint infections.

These results are in accordance with the literature, as CNS are considered the predominantly involved microorganisms in PJIs with a range in the detection rate of 27–75% [17, 18]. Regarding infections of native joints, *S. aureus* is a main causative in about 39–60% of cases [19, 20]. Despite the existence of these data being in line with the results of the present study, there is also some contradictory evidence for a prevalent role of *S. aureus* in PJIs [21]. Siu et al. reported a 44.1% detection rate of *S. aureus* in 34 PJIs, of which 70% were late-onset infections, without specific cause clarification. The data situation regarding infections with Enterobacterales in native joints is still indecisive, whereas a detection rate of 15% is reported for implant associated joint infections [22]. In the present study, a large proportion of 66.1% of CNS were oxacillin-resistant in PJIs, and the literature describes rates of up to 77.8% [22]. However, most of these studies were not primarily designed for detecting variations in the pathogen spectrum between the two groups.

Indeed, several studies observed that pre-existing comorbidities, such as diabetes mellitus and renal insufficiency, increase the risk of infection after joint replacement, as well as mortality [23, 24]. Evidence for differing pathogen spectra depending on the presence of these diseases is weak. Our study results indicate a similar bacterial distribution in joint infections of patients with or without renal insufficiency or diabetes mellitus.

While detecting potentially causative bacteria in joint infections is a key element in diagnostics, in addition to a proper surgical therapy, the patients’ outcome largely depends on an adequate antimicrobial drug therapy. Early empiric antibiotic therapy is therefore considered a significant component in the treatment of both, NJIs and PJIs [4, 25]. However, due to the heterogeneity of medical treatment concepts, evidence for a superior effectiveness of one specific calculated antibiotic regime in NJIs is lacking [25, 26]. Regarding early antibiotic therapy in infections of prosthetic joints, the literature states diverse approaches as well [27, 28]. Despite the knowledge of predominantly involved bacteria in PJIs and NJIs, consistent international guidelines for early antibiotic therapy are still missing.

Notably, CNS and *S. aureus* are the predominant pathogen types in both, periprosthetic and native joint infections. As PJI and NJI patients are usually initially treated by the same empiric antibiotic regime, the presence or absence of a joint prosthesis seems therefore not to be relevant with respect to early calculated anti-infective drug therapy concepts.

Nevertheless, novel aspects emerge from this current study. With a detection rate of 18.8% of Enterobacterales, their importance in PJIs may have been underestimated yet. Thus, it may be an option also to include antibiotics targeting gram-negative bacteria in early antibiotic therapy in PJI. In addition, we found that 2/3 of CNS in prosthetic joint infections were oxacillin-resistant. Consequently, broad-spectrum coverage of these pathogens in empiric anti-infective medication, such as with vancomycin in the absence of contraindications, should be considered.

This study has some limitations. Regarding PJIs, we did not consider whether the synovial fluid analysis was performed in an acute or chronic infection, although differing pathogen spectra are known for these subgroups [3]. Furthermore, in NJIs, the primary focus of infection was not evaluated and possible differences in the microbial spectrum of iatrogenic, post-traumatic or haematogenous infections remain therefore uncertain. Samples were collected from closed joint aspirates only, and intraoperative culture results were not included in the microbiological analyses. Hence, there is a risk that the totality of pathogens actually involved in joint infections is not entirely represented in the study results. Finally, as this is a retrospective study, it is limited by its inherent methodological drawback.

## Conclusion

*Staphylococcus aureus* and CNS are the predominantly involved microorganisms in PJIs and NJIs. Nevertheless, the high rate of oxacillin resistances in CNS, as well as the elevated detection rate of Enterobacterales, may necessitate a change in early antibiotic therapy in PJIs.

## Data Availability

The datasets used and analyzed during the current study are available from the corresponding author on reasonable request.

## Abbreviations

PJI: Periprosthetic joint infections
NJI: Native joint infections
CNS: Coagulase-negative *Staphylococci*
*S. aureus*: *Staphylococcus aureus*
*S. epidermidis*: *Staphylococcus epidermidis*
*S. capitis*: *Staphylococcus* *capitis*
*S. hominis*: *Staphylococcus* *hominis*
*S. lugdunensis*: *Staphylococcus* *lugdunensis*
*S. caprae*: *Staphylococcus caprae*
*E. coli*: *Escherichia coli*
MRSA: Oxacillin-resistant *S. aureus*
